# Functional and Structural Insights into Lipases Associated with Fruit Lipid Accumulation in *Swida wilsoniana*

**DOI:** 10.3390/biom16010092

**Published:** 2026-01-06

**Authors:** Wei Wu, Yunzhu Chen, Changzhu Li, Peiwang Li, Yan Yang, Lijuan Jiang, Wenyan Yuan, Qiang Liu, Li Li, Wenbin Zeng, Xiao Zhou, Jingzhen Chen

**Affiliations:** 1 State Key Laboratory of Woody Oil Resources Utilization, Hunan Academy of Forestry, Changsha 410004, China; 20221200173@csuft.edu.cn (W.W.); cyzcarol@163.com (Y.C.); lichangzhu2013@aliyun.com (C.L.);; 2 College of Life Science and Technology, Central South University of Forestry and Technology, 498 South Shaoshan Road, Changsha 410004, China

**Keywords:** lipase, *Swida wilsoniana*, molecular docking, lipid metabolism

## Abstract

*Swida wilsoniana* is an important oil-producing tree species whose fruits are rich in unsaturated fatty acids with high nutritional and medicinal value. Lipases are involved not only in lipid mobilization but also potentially in the regulation of fatty acid composition and oil accumulation in plants. In this study, the fatty acid composition of *S. wilsoniana* fruits was analyzed using gas chromatography–flame ionization detection (GC-FID), and the three most abundant fatty acids were selected as molecular docking ligands. Based on overall multi-ligand docking performance (including mean affinity across the three ligands), three key lipases—SwL5, SwL8, and SwL12—were identified as having the strongest interactions with these fatty acids. Phylogenetic analysis revealed that SwL5 and SwL12 belong to lipase family II, while SwL8 is classified into family VI. Molecular dynamics simulations were further performed to evaluate the binding stability and to characterize the structural basis of substrate recognition, including key interacting residues. This study provides theoretical insights into the molecular regulation of fatty acid composition in *S. wilsoniana*, and offers potential gene targets for the genetic improvement of oil quality traits.

## 1. Introduction

*Swida wilsoniana* is a woody oil-bearing species with significant development potential, widely distributed in southern China [1]. Its fruits exhibit a relatively high oil content and are rich in unsaturated fatty acids such as oleic acid and linoleic acid, making them promising for the development of functional and high-quality plant oils [2,3]. In recent years, growing market demand for oils with enhanced nutritional properties and greater fatty acid diversity has elevated the importance of *S. wilsoniana* as a novel oil source [4]. With breeding targets shifting from “high yield” to “high quality,” studies focusing on the mechanisms of oil biosynthesis and accumulation have received increasing attention [5,6,7]. However, systematic studies on oil-related genes in *S. wilsoniana* remain limited, and key enzyme resources involved in regulating oil composition have not yet been clearly identified, which has hindered progress in its genetic improvement. Thus, identifying core regulators involved in oil quality formation is of great significance for trait-oriented breeding. Lipases, as enzymes involved in lipid metabolism, have been found to participate in early-stage lipid biosynthesis in several plant species, contributing to fatty acid recognition and reaction specificity [8,9,10]. Some members of the GDSL lipase family have been shown in model species such as *Arabidopsis* to influence seed oil content and fatty acid composition, suggesting that their broader functional potential in non-model oil plants remains underexplored [11,12,13]. Although a few lipases have been functionally characterized, their structural basis, substrate preferences, and roles in oil composition formation are still poorly understood in woody species like *S. wilsoniana* [14,15]. Integrating structural modeling, computational screening, and experimental validation could provide a useful means of identifying their possible roles.

In this study, genome annotation and fruit fatty acid composition data of *S. wilsoniana* were used to investigate the binding characteristics between lipases and major fatty acid molecules, with the goal of identifying candidate lipase genes potentially involved in oil storage regulation. Molecular docking and molecular dynamics simulations were conducted to analyze the stability and recognition mechanisms of lipase–fatty acid complexes. This study provides a theoretical foundation for understanding the molecular regulation of fatty acid composition in *S. wilsoniana* and offers potential targets for the genetic improvement of oil quality traits. It also provides a methodological framework that may be extended to other underutilized woody oil plants.

## 2. Materials and Methods

### 2.1. Materials

The biological material used in this study consisted of fruits of *Swida wilsoniana*, collected from the National Tree Breeding Base at the Experimental Forest Farm of the Hunan Academy of Forestry. Following collection, the samples were promptly snap-frozen using liquid nitrogen and preserved at −80 °C until further analysis. The PrimeScript™ RT reagent Kit (with gDNA Eraser) and the TB Green^®^ Premix Ex Taq™ II were supplied by Takara, Japan (Kyoto). Total RNA extraction was carried out using the RNAprep Pure Plant Kit (TIANGEN Biotech, Beijing, China). Standards of fatty acids were sourced from Sigma-Aldrich, St. Louis, MI, USA. Methanol, acetonitrile, and formic acid of chromatographic purity were purchased from Merck (Darmstadt, Germany), while all additional reagents used in the experiments were either analytical grade or suitable for molecular biology applications.

The primary analytical instruments included a Nexis GC-2030 gas chromatograph equipped with a flame ionization detector (Shimadzu, Kyoto, Japan), a CFX96 real-time quantitative PCR platform (Bio-Rad, Hercules, CA, USA), an Epoch 2 microplate reader (BioTek Instruments, Winooski, Vermont, USA), and a 5424 R high-speed refrigerated centrifuge (Eppendorf, Hamburg, Germany). Additional standard laboratory instruments were employed as required.

### 2.2. Extraction and Methylation of Fatty Acids

Fatty acid methyl esters (FAMEs) were prepared from *Swida wilsoniana* fruit oil using a base-catalyzed methylation procedure. Briefly, approximately 0.06 g of oil was placed into a 10.0 mL centrifuge tube, followed by addition of 4.0 mL isooctane. Subsequently, 2.0 mL of KOH–methanol solution (2 mol/L) was added, and the mixture was vigorously shaken for 60 s. Then, 1.0 g of anhydrous NaHSO_4_ was added, and the tube was shaken for another 30 s and allowed to stand for 10 min. The upper organic phase was collected, filtered through a 0.22 μm organic membrane, and subjected to GC analysis.

### 2.3. Gas Chromatography (GC-FID)

FAMEs were analyzed on a Nexis GC-2030 gas chromatograph (Shimadzu, Kyoto, Japan) equipped with a flame ionization detector (FID) and a fused-silica capillary column (0.25 mm × 100 m, 0.25 μm film thickness). Nitrogen was used as the carrier gas at a flow rate of 1.1 mL/min, with a split ratio of 1:100. The oven temperature program was set as follows: initial 100 °C for 13 min; 100–180 °C at 10 °C/min and held for 6 min; 180–200 °C at 1 °C/min and held for 20 min; and 200–230 °C at 4 °C/min and held for 10.5 min. The injector temperature was 270 °C and the injection volume was 1.0 μL. Fatty acids were identified by comparing retention times with Supelco 37 Component FAME Mix (Supelco: CRM47885, Bellefonte, PA, USA). Relative fatty acid contents were calculated by peak-area normalization to the total chromatographic peak area. The raw GC chromatograms and peak integration tables from the GC experiment are provided in the Supplementary Document.

### 2.4. Quantitative Real-Time PCR

Total RNA was isolated using the RNAprep Pure Plant Kit, and cDNA was synthesized via reverse transcription with the PrimeScript™ RT reagent Kit. Quantitative PCR analysis was carried out on the CFX96 Real-Time PCR System (Bio-Rad) using TB Green^®^ Premix Ex Taq™ II. RNA concentration and purity were assessed using a spectrophotometer. The extracted RNA showed a concentration of 67.835 ng/µL and an A260/280 ratio of 2.084. Each 20 μL reaction consisted of 10 μL of 2× master mix, 0.4 μL of each primer (10 μM), 2 μL of cDNA, and nuclease-free water.

Cycling parameters included an initial denaturation at 95 °C for 30 s, followed by 40 cycles of 95 °C for 5 s and 60 °C for 30 s. Reactions were performed in triplicate for each biological sample. Relative transcript levels were calculated using the 2^−ΔΔCt^ method [16]. Ct values of target genes were normalized against UBC (ubiquitin-conjugating enzyme) as the internal reference gene, and root tissue was used as the calibrator. Primer sequences for SwL5, SwL8, SwL12, and UBC are provided in Supplementary Table S1.

### 2.5. Identification and Filtering of Key Lipase Genes

The whole-genome data of *Swida wilsoniana* used in this study were obtained from our previously conducted genome sequencing and assembly project [17]. Based on the functional annotation results from the NR database, protein-coding sequences with the keyword “lipase” in their functional descriptions were initially screened. Sequences clearly misannotated as transcription factors, transporters, or other unrelated proteins were removed, and a set of candidate genes with lipase-related functions was obtained.

Candidate protein sequences were clustered using CD-HIT version 4.8.1 with a 95% identity cutoff, and representative non-redundant sequences were selected for subsequent analyses. To clarify their structural characteristics and catalytic potential, protein domain prediction was performed using the InterPro database. Sequences lacking typical lipase-associated domains or missing conserved catalytic triad residues were filtered out. Finally, lipase genes with complete domain structures and reliable annotations were retained for downstream analyses.

### 2.6. Homology Modeling and Molecular Docking

To investigate the binding characteristics between *Swida wilsoniana* lipases and major fatty acids, the three most abundant fatty acids in the fruit were selected as ligand molecules and individually docked with the candidate lipase proteins. Homology models of lipase proteins were generated using the SWISS-MODEL online platform (https://swissmodel.expasy.org/ (accessed on 18 September 2025)) and validated by Ramachandran plot analysis via the SAVES 6.1 server (https://saves.mbi.ucla.edu/ (accessed on 21 September 2025)). The molecular structures of fatty acids were retrieved from the PubChem database (https://pubchem.ncbi.nlm.nih.gov/ (accessed on 29 September 2025)).

Molecular docking was performed using AutoDock Vina (v1.1.2) [18]. Both receptor and ligand molecules were preprocessed with AutoDockTools, including hydrogen addition, Gasteiger charge assignment, and conversion to pdbqt format. The docking grid box was set to 50 × 50 × 50 Å, and the grid center was automatically adjusted based on the protein structure to ensure full surface coverage and comprehensive binding site sampling. For each fatty acid, the lipase with the lowest binding affinity (most stable interaction) was selected for further analysis. Candidates were prioritized using a multi-ligand (consensus) docking rationale across linoleic, oleic, and palmitic acids.

The docking conformations and spatial orientations were visualized using Visual Molecular Dynamics (VMD, v1.9.2), and intermolecular interactions (such as hydrogen bonds and hydrophobic contacts) were automatically analyzed and illustrated using LigPlot+ (v2.2.9) [19].

### 2.7. Phylogenetic Analysis

Phylogenetic analysis was conducted using their protein sequences together with 28 reference sequences representing eight plant lipase families. Reference sequences were retrieved from the NCBI database and manually curated to ensure accurate annotation and structural completeness.

Protein sequences were aligned using the ClustalW algorithm implemented in MEGA 11 with standard settings. A phylogenetic tree was generated through the Maximum Likelihood approach based on the JTT model, and node confidence was assessed via 1000 bootstrap replicates.

### 2.8. Molecular Dynamics Simulation

Molecular dynamics simulations were performed using the GROMACS 2020.6 platform [20]. The CHARMM36 force field was applied to the protein, and topology files for the fatty acid ligands were generated using the CGenFF 3.0.1 webserver (https://cgenff.com (accessed on 6 October 2025)). Each lipase–ligand complex was embedded in an orthorhombic box filled with TIP3P water. Counterions (Na^+^ and Cl^−^) were subsequently introduced to ensure system neutrality. Energy minimization was conducted for 5000 steps using the steepest descent method. Subsequently, the system was equilibrated under constant temperature (NVT) and constant pressure (NPT) ensembles for 100 picoseconds each, with temperature and pressure maintained at 300 K and 1 atm, respectively. A 100-nanosecond production run was then carried out with a 2-femtosecond integration step. Bond constraints were applied using the LINCS algorithm, while long-range electrostatics were treated via the particle mesh Ewald (PME) approach.

## 3. Results

### 3.1. Fatty Acid Profile Characteristics of Swida wilsoniana Oil

**Figure 1:**
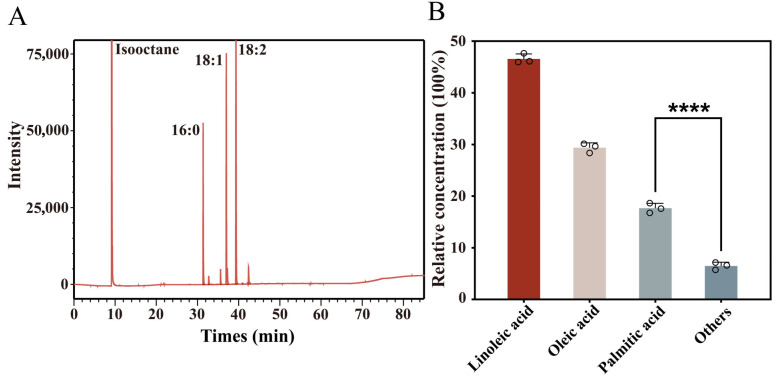
GC-FID analysis of fatty acid composition in *Swida wilsoniana* oil. (**A**) GC-FID chromatogram of fatty acid methyl esters showing peak profiles of major constituents, including linoleic acid (18:2), oleic acid (18:1), and palmitic acid (16:0). (**B**) Relative contents (%) of different fatty acids calculated by normalization to total peak area (Significance is indicated as follows: **** *p* < 0.0001).

The qualitative and quantitative composition of major fatty acids in *S. wilsoniana* fruit was examined using GC-FID analysis to explore their accumulation profiles. As shown in the GC chromatogram in Figure 1A, the main fatty acid methyl ester peaks were well resolved, with strong signals corresponding to 16:0 (palmitic acid), 18:1 (oleic acid), and 18:2 (linoleic acid), indicating their high abundance in the sample.

Relative quantification based on normalized peak areas (Figure 1B) revealed that linoleic acid was the most abundant component, accounting for approximately 46.4% of total fatty acids, followed by oleic acid (29.9%) and palmitic acid (17.3%), while all other fatty acids together contributed less than 7%. In the GC chromatogram, the prominent peak around 10 min corresponds to the isooctane solvent and was not included in peak integration for FAME quantification. Linoleic and oleic acids are typical C18 unsaturated fatty acids known for their nutritional and physiological value, whereas palmitic acid, a saturated fatty acid, serves as a key intermediate in fatty acid biosynthesis [21,22]. These results suggest that the oil in *S. wilsoniana* fruit is predominantly composed of C18 unsaturated fatty acids, exhibiting the typical profile of high-quality plant oils.

Based on their dominant abundance, linoleic acid (18:2), oleic acid (18:1), and palmitic acid (16:0) were used as ligands for subsequent molecular docking analyses.

### 3.2. Identification of Candidate Lipases

To identify lipase genes potentially involved in lipid metabolism regulation in *Swida wilsoniana*, a total of 100 putative lipase-related genes were initially screened based on NR database annotations of the whole genome. After domain conservation analysis and functional curation, 18 candidate genes containing typical lipase domains were retained, and both the genes and their encoded enzymes were designated as SwL1 to SwL18 (Table 1).

The three predominant fatty acids in *S. wilsoniana* oil—linoleic, oleic, and palmitic acids—were chosen as ligand molecules to assess substrate recognition preferences. Each candidate lipase was subjected to molecular docking with these ligands, and candidates were prioritized using a multi-ligand (consensus) docking rationale based on their overall performance across the three ligands, as reflected by the mean affinity across the three ligands.

The results revealed distinct binding preferences among the candidate genes (Table 1). SwL5 exhibited the strongest binding to palmitic acid (−5.9 kcal/mol), SwL8 had the lowest binding energy with linoleic acid (−7.3 kcal/mol), and SwL12 showed the highest affinity for oleic acid (−6.7 kcal/mol). These three genes demonstrated clear substrate preferences and were selected for subsequent structural visualization and molecular dynamics simulations. Notably, SwL8, SwL5, and SwL12 were the top three candidates by mean affinity across the three ligands, indicating the most favorable overall multi-ligand binding profiles among the tested variants.

### 3.3. Tissue-Specific Expression Patterns of the Key Lipase Genes

To better understand the functional roles of key lipase genes, qPCR was performed to assess the expression levels of SwL5, SwL8, and SwL12 in various tissues of *S. wilsoniana*, including root, pulp, stem, and leaf. All three genes exhibited significant tissue-specific expression patterns, with the highest transcript abundance detected in the pulp.

**Figure 2:**
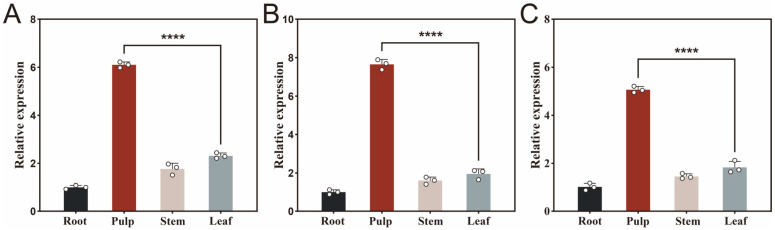
Relative expression levels of three key lipase genes in different tissues of *S. wilsoniana*. Panels (**A**–**C**) show the qPCR results of SwL5 (**A**), SwL8 (**B**), and SwL12 (**C**) in root, pulp, stem, and leaf tissues. Relative expression levels were normalized to UBC (reference gene), with root used as the calibrator. Asterisks indicate statistically significant differences compared to root (**** *p* < 0.0001, two-tailed *t*-test).

*SwL5* showed approximately 6.2 fold higher expression in the pulp compared to root, and also elevated levels in the stem and leaf (Figure 2A), suggesting its involvement in lipid metabolism across multiple tissues, particularly in the fruit. *SwL8* exhibited the strongest expression in the pulp, about 7.8 fold relative to root (Figure 2B), which aligns well with its highest binding affinity toward linoleic acid. This indicates that *SwL8* may act as a major lipase during fatty acid accumulation in fruit development. In contrast, *SwL12* showed a 5.1 fold increase in pulp compared to root (Figure 2C), which, although lower than the other two, still suggests a potential role in oleic acid recognition and modification during late-stage lipid biosynthesis.

### 3.4. Phylogenetic Analysis of Key Lipases

To further determine the family classification and evolutionary relationships of the three selected key lipases, 28 well-annotated reference lipase protein sequences from various species were selected, representing eight distinct families (I–VIII), for phylogenetic tree construction.

**Figure 3:**
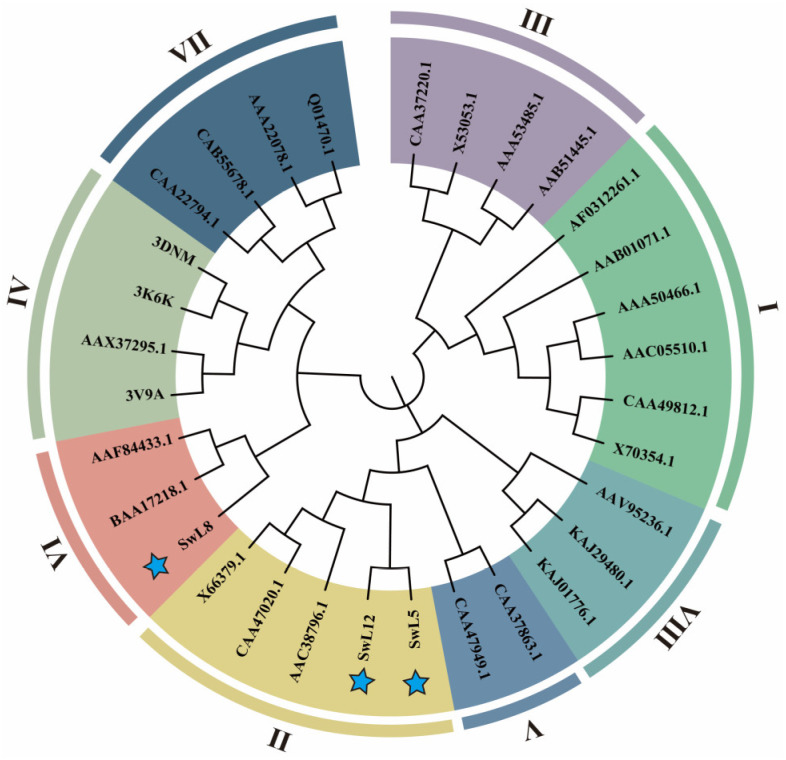
Phylogenetic analysis of three key lipases. A maximum likelihood phylogenetic tree was constructed using SwL5, SwL8, and SwL12 along with 28 reference lipases from eight distinct families (I–VIII). Colored segments indicate family classifications. Blue stars denote the three candidate lipase genes identified in this study.

The resulting phylogenetic tree revealed that SwL5 and SwL12 belong to Family II, while SwL8 clusters within Family VI (Figure 3). These results suggest a clear structural and evolutionary divergence among the three genes, indicating that they may play distinct roles in fatty acid recognition, transport, or storage regulation in *Swida wilsoniana* [23].

### 3.5. Three-Dimensional Structure Prediction and Conformational Validation of Key Lipases

**Figure 4:**
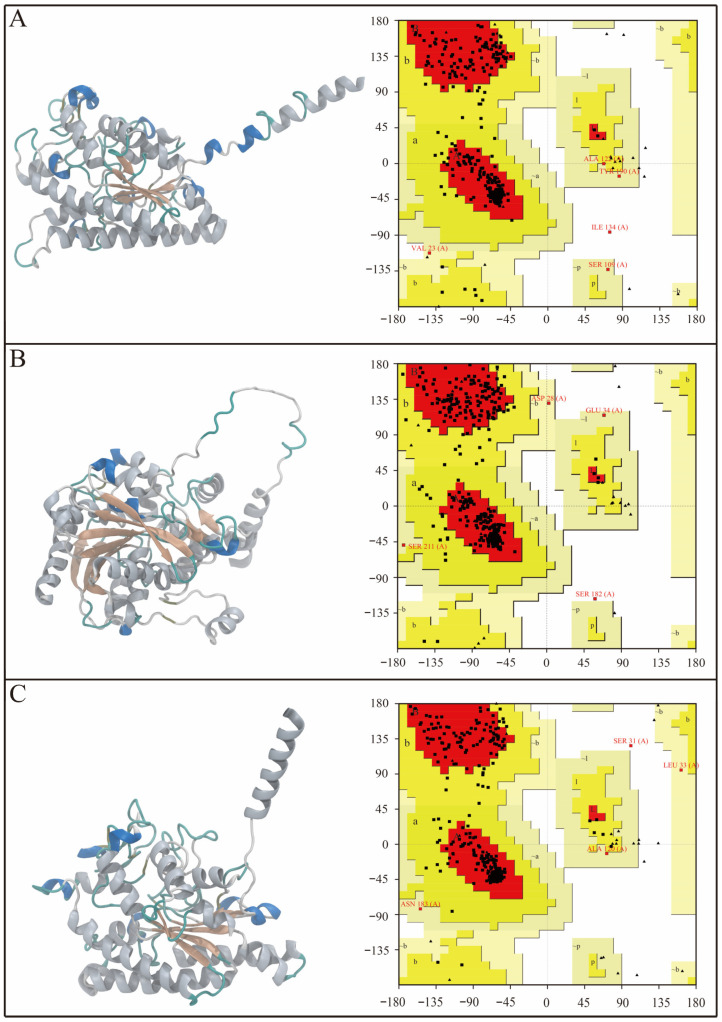
Predicted 3D structures and Ramachandran plot evaluation of SwL5, SwL8, and SwL12. Panels (**A**–**C**) show the homology-modeled structures (**left**) and corresponding Ramachandran plots (**right**) of SwL5, SwL8, and SwL12, respectively. Structures were modeled using SWISS-MODEL, and plots were generated with SAVES 6.0.

To obtain the spatial conformations of the candidate lipases for molecular docking and simulation, homology models of SwL5, SwL8, and SwL12 were generated using the SWISS-MODEL server. As illustrated in Figure 4 (left panels), all three predicted proteins adopt the canonical α/β-hydrolase fold, consisting of a central β-sheet flanked by surrounding α-helices, which is a common feature of lipase structures and suggests proper tertiary folding and catalytic competence [24].

To further validate the stereochemical quality of these models, Ramachandran plots were generated using SAVES 6.0. As shown in Figure 4 (right panels), over 90% of residues in each model were located in favored regions, with the remainder mostly in allowed regions and only a small number of outliers [25]. These results indicate that the models were structurally sound and suitable for subsequent molecular docking and dynamics simulations.

### 3.6. Molecular Docking Analysis

To investigate the substrate recognition characteristics of key lipases, three major fatty acids identified from *S. wilsoniana* fruit—palmitic acid (C16:0), linoleic acid (C18:2), and oleic acid (C18:1)—were used as ligand molecules for docking with SwL5, SwL8, and SwL12, respectively. The results are shown in Figure 4.

**Figure 5:**
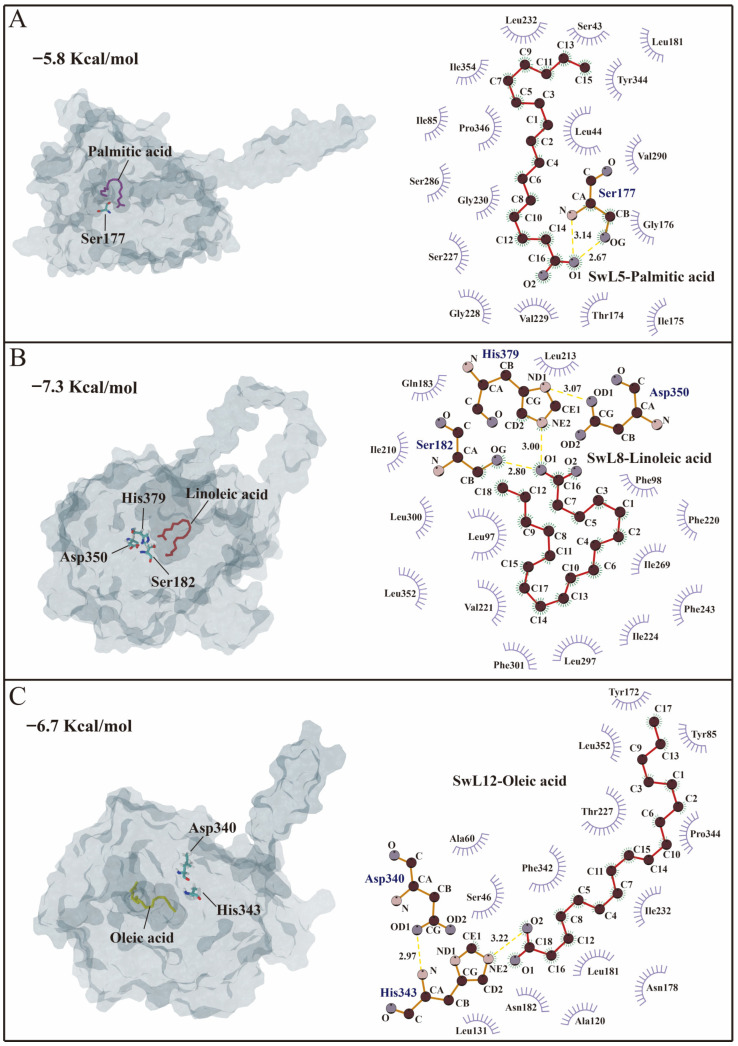
Molecular docking conformations and interaction analysis of SwL5, SwL8, and SwL12 with major fatty acids. Panels (**A**–**C**) show the docking results of SwL5–palmitic acid, SwL8–linoleic acid, and SwL12–oleic acid, respectively. (**Left**) 3D binding conformations; (**Right**) 2D interaction maps generated by LigPlot^+^. The three lipases exhibit distinct binding patterns and key interacting residues, indicating specific substrate recognition mechanisms.

SwL5 was docked with palmitic acid, resulting in a binding affinity of −5.8 kcal/mol (Figure 5A). The fatty acid was accommodated within a relatively open hydrophobic pocket. Two hydrogen bonds were formed between the carboxyl group of palmitic acid and Ser177, with bond lengths of 2.67 Å and 3.14 Å. Additional hydrophobic interactions with residues such as Gly176, Val229, and Leu232 further stabilized the binding pose, indicating a potential role of SwL5 in recognizing saturated medium-chain fatty acids.

SwL8 exhibited the strongest binding with linoleic acid, with a binding energy of −7.3 kcal/mol (Figure 5B). The ligand formed two hydrogen bonds with Ser182 and His379, two members of the catalytic triad (Ser182, Asp350, His379), at 3.00 Å and 2.80 Å, respectively. An additional hydrogen bond of 3.07 Å was formed between His379 and Asp350, stabilizing the catalytic site. This configuration suggests a classic triad-based recognition mode, highlighting the potential involvement of SwL8 in the selective binding and regulation of polyunsaturated fatty acid accumulation [26,27].

SwL12 showed a binding affinity of −6.7 kcal/mol with oleic acid (Figure 5C). A hydrogen bond was observed between the fatty acid and His343 (2.91 Å), and another internal hydrogen bond was formed between His343 and Asp340 (3.22 Å), contributing to the stability of the active pocket [28,29]. Hydrophobic residues surrounding the cavity, such as Leu131, Phe342, and Tyr172, may enhance the affinity for long-chain monounsaturated fatty acids [30].

### 3.7. Analysis of Molecular Dynamics Simulation Results

**Figure 6:**
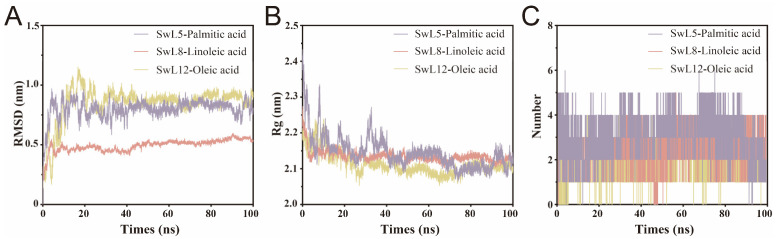
Global Structural Stability Analysis of Lipase–Fatty Acid Complexes. (**A**) Backbone root-mean-square deviation of each complex over the 100 ns molecular dynamics simulation; (**B**) Radius of gyration profiles indicating structural compactness; (**C**) Number of hydrogen bonds formed between lipase and fatty acid during the simulation. The three complexes analyzed include SwL5–palmitic acid, SwL8–linoleic acid, and SwL12–oleic acid.

To investigate the conformational stability of lipase-fatty acid complexes during simulation, root-mean-square deviation (RMSD), radius of gyration (Rg), and hydrogen bond number were analyzed over a 100 ns trajectory (Figure 6A–C).

SwL8-linoleic acid complex exhibited the most stable behavior, with RMSD and Rg values reaching equilibrium within 10 ns. The RMSD stabilized at approximately 0.5 nm, remaining consistently lower than that of the other two complexes. This early stabilization suggests a compact and rigid structural conformation upon binding linoleic acid, which is consistent with its lowest binding energy among the three fatty acids [31].

Similarly, SwL12-oleic acid complex achieved RMSD equilibrium at around 10 ns, but Rg required nearly 40 ns to stabilize. The final RMSD was higher than SwL8-linoleic acid (1.0 nm), indicating relatively greater structural fluctuation during the simulation [32,33].

In contrast, SwL5-palmitic acid complex, which had the highest binding energy, showed delayed structural stabilization. Both RMSD and Rg reached equilibrium at approximately 30 ns, with the RMSD level comparable to that of SwL12-oleic acid. Interestingly, SwL5-palmitic acid displayed the lowest Rg value after stabilization, suggesting a more compact conformation despite its higher flexibility earlier in the simulation [34].

Hydrogen bond analysis revealed an intriguing contradiction. Although the SwL5–palmitic acid complex consistently exhibited the highest number of hydrogen bonds, its RMSD and Rg took longer to stabilize and showed greater fluctuations, suggesting that hydrogen bond abundance alone does not confer structural stability [35]. These interactions may be less persistent or poorly organized, reflecting conformational rearrangements or internal flexibility [36]. In contrast, the SwL8–linoleic acid complex, despite forming fewer hydrogen bonds, exhibited the earliest convergence and lowest RMSD values, indicating a more compact and stable binding mode [37]. This highlights the importance of interaction quality and spatial coherence over quantity in governing dynamic stability.

To further investigate the local flexibility of each lipase in the presence of different fatty acids, the root-mean-square fluctuation (RMSF) values of all residues were calculated across the 100 ns simulation.

**Figure 7:**
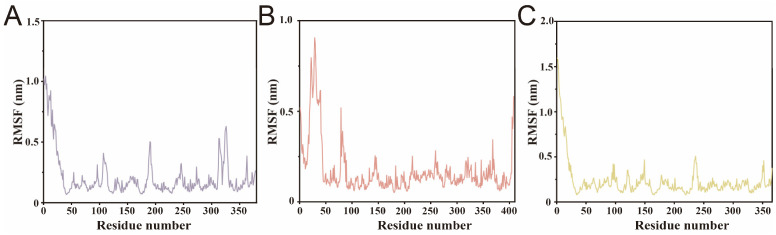
Residue Flexibility Analysis of Lipase–Fatty Acid Complexes. Panels (**A**–**C**) show the root-mean-square fluctuation (RMSF) profiles of SwL5–palmitic acid, SwL8–linoleic acid, and SwL12–oleic acid complexes in order.

SwL12–oleic acid complex exhibited the highest overall RMSF values, indicating greater local flexibility throughout the protein structure (Figure 7A) [38]. This was followed by SwL5–palmitic acid, which also showed several fluctuating regions, particularly in loop areas (Figure 7B) [39]. In contrast, SwL8–linoleic acid displayed the lowest RMSF across most residues, suggesting a more rigid protein conformation upon ligand binding (Figure 7C). Notably, all three complexes showed a prominent flexible region at the N-terminus, likely corresponding to an unstructured loop or terminal extension [40]. The variation in residue-level mobility is consistent with the earlier RMSD/Rg observations, further supporting that SwL8 binding stabilizes the protein more effectively.

To further explore the conformational dynamics of lipase–fatty acid complexes, structural snapshots were extracted at 25 ns intervals throughout the 100 ns simulation. All three systems displayed progressive structural compaction, consistent with the overall folding trend observed in water-box views.

**Figure 8:**
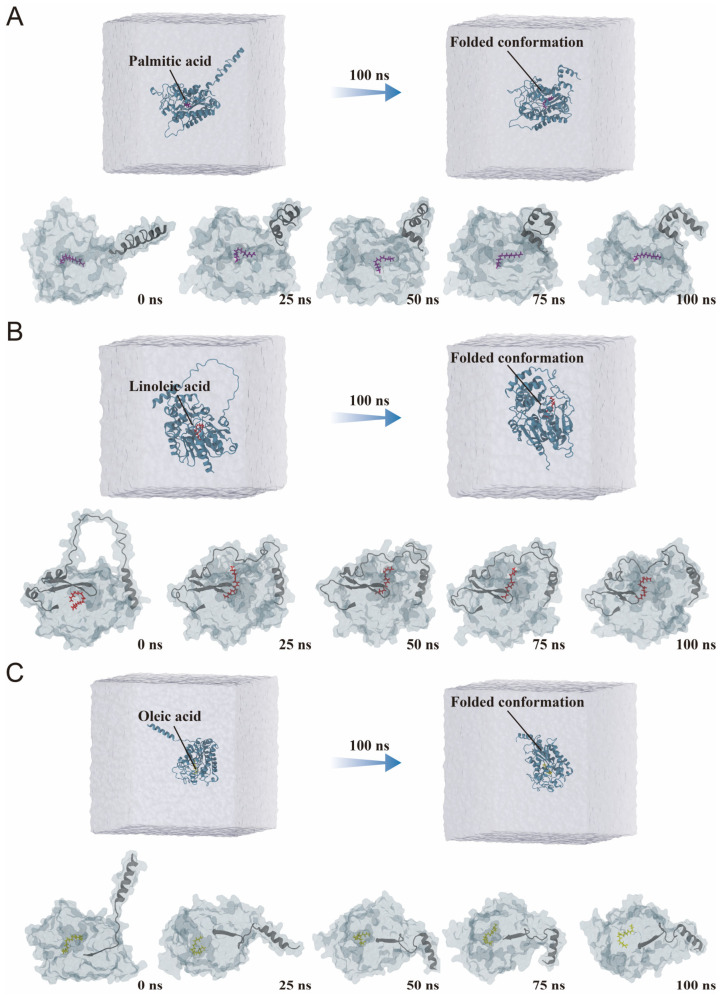
Conformational evolution of lipase–fatty acid complexes during molecular dynamics simulations. (**A**) SwL5–palmitic acid complex; (**B**) SwL8–linoleic acid complex; (**C**) SwL12–oleic acid complex. The (**upper panels**) show the overall structures of the complexes within the solvent box at the beginning (0 ns) and end (100 ns) of the simulation. The (**lower panels**) display conformational snapshots of the protein–ligand complexes taken every 25 ns, illustrating their dynamic structural transitions.

The SwL5–palmitic acid complex underwent the most pronounced conformational fluctuation. It exhibited a distinct “folding–unfolding” transition, indicative of structural instability during the simulation (Figure 8A). The ligand position also showed signs of displacement, suggesting weaker or less stable interactions. The SwL8–linoleic acid complex displayed an initial expansion of its structure, followed by a mild refolding phase (Figure 8B). This may reflect an adaptive binding adjustment and is consistent with its high rigidity observed in RMSF results. In contrast, the SwL12–oleic acid complex showed a continuous and smooth folding trajectory (Figure 8C). The ligand gradually migrated into the catalytic cavity, maintaining proximity to key residues throughout the simulation [41]. This behavior suggests a well-organized and stable binding mode with minimal structural disruption [42].

## 4. Discussion

This study integrates fatty acid profiling with structure-based docking and molecular dynamics simulations to prioritize lipase candidates that may be involved in shaping oil composition in *S. wilsoniana*. In this section, we interpret the lipid profile together with the computational analyses to formulate mechanistic hypotheses for the selected candidates. We emphasize that docking-derived affinity values are not direct proxies for in vivo contribution, because physiological relevance also depends on factors such as expression level, subcellular localization, and developmental context. Accordingly, docking was used here as an in silico prioritization approach to identify candidates with a balanced binding profile across the predominant fatty acids, while the pulp-enriched expression observed for SwL5/SwL8/SwL12 provides supportive context consistent with their potential involvement in fruit lipid accumulation.

The lipid profile of *S. wilsoniana* fruit oil is dominated by three fatty acids—linoleic acid (18:2), oleic acid (18:1), and palmitic acid (16:0)—with C18 unsaturated fatty acids constituting the major fraction. This compositional pattern not only highlights the nutritional relevance of *S. wilsoniana* oil, but also provides a biologically grounded context for probing lipid-metabolism enzymes that might influence fatty-acid partitioning during fruit development. Therefore, these predominant fatty acids were used as representative ligands to examine whether any lipase variants show consistent recognition trends across the major oil components, thereby informing downstream structural analyses.

Importantly, our candidate selection was not based on a single ligand-specific docking score. Although some unselected variants show docking affinities close to the selected ones for an individual ligand, only SwL5, SwL8, and SwL12 maintained comparatively favorable affinities across all three predominant fatty acids, reflecting the most balanced multi-ligand binding profiles among the tested variants (Table 1). This “multi-ligand consistency” rationale is further captured by the mean affinity across the three ligands, where SwL8, SwL5, and SwL12 rank among the top candidates. In this sense, docking was used to prioritize candidates with broad compatibility to the oil’s dominant substrates, rather than to assert physiological dominance solely from affinity values. A docking study reported binding energies between −5 and −7 kcal/mol for a lipase in complex with oleic acid and with tripalmitin, and the energies observed here, from −5.8 to −7.3 kcal/mol, are in the same magnitude range, supporting the plausibility of the predicted interactions rather than scoring artifacts [43].

At a structural level, differential binding behaviors among lipases are plausibly influenced by active-site architecture, spatial distribution of key residues, and hydrophobic microenvironments42, 43. The distinct binding conformations and interaction networks observed for SwL5, SwL8, and SwL12 support the interpretation that these enzymes may differ in substrate accommodation and recognition. Such structural variation provides a mechanistic basis for functional divergence, and it motivates further analysis of how catalytic-site geometry and pocket physicochemical properties may collectively shape substrate preference. The amino acid sequences of these three key lipases are provided in the Supplementary Document to facilitate future experimental validation and comparative analyses. In addition, the corresponding sequences have been deposited in NCBI GenBank under accession numbers PX561283 (SwL5), PX561284 (SwL8), and PX561285 (SwL12).

Molecular dynamics simulations add an additional layer of evidence beyond static docking snapshots. Across the trajectories, the complexes exhibited distinct patterns of conformational convergence, compactness, and intermolecular interaction coherence, indicating that docking affinity alone is not a definitive predictor of dynamic stability. In particular, the SwL8–linoleic acid complex showed the most rapid and stable convergence, consistent with a more coherent and stable binding mode, whereas other complexes displayed greater structural fluctuation despite in some cases forming more hydrogen bonds. Together, these results suggest that interaction quality and spatial organization—not merely the number of contacts—may govern dynamic stability, with SwL8–linoleic acid emerging as the most stable and functionally promising complex in our simulations. Consistent with this, RMSF patterns indicate that linoleic acid binding is associated with reduced local fluctuations, supporting tighter ligand–protein coupling and a more rigid complex conformation.

Although the current evidence is primarily computational, the prioritized candidates (SwL5, SwL8, and SwL12) provide a focused set of targets for experimental validation, including in vitro activity assays with defined fatty-acid substrates, subcellular localization analyses, and gain-/loss-of-function studies during fruit development. Such follow-up experiments will be essential to establish causal relationships between lipase activity, fatty-acid composition, and oil quality traits, and to determine whether the predicted substrate preferences and stability differences translate into measurable biochemical functions in vivo.

The differential binding behaviors of lipases to fatty acids are likely influenced by factors such as active site architecture, spatial distribution of key amino acid residues, and hydrophobic microenvironments [44,45]. Detailed structural analyses of SwL5, SwL8, and SwL12 will provide insight into their potential roles in determining the oil composition of *S. wilsoniana*, and offer theoretical support for functional validation and targeted improvement of oil quality traits. The amino acid sequences of these three key lipases can be found in the Supplementary Document. Detailed structural analyses of SwL5, SwL8, and SwL12 will provide insight into their potential roles in determining the oil composition of S. wilsoniana, and offer theoretical support for functional validation and targeted improvement of oil quality traits. The amino acid sequences of these three key lipases can be found in the Supplementary Document.

In summary, the high expression of these three genes in the pulp correlates with the predominant oil accumulation in this tissue, reinforcing their possible contribution to fruit lipid metabolism. These findings provide molecular evidence for their functional relevance and offer candidate targets for further genetic improvement of oil traits in *S. wilsoniana*.

This analysis provides evolutionary support for functional annotation and lays a theoretical foundation for understanding the functional diversity of lipases in *S. wilsoniana*.

In summary, the three lipases displayed distinct differences in binding conformation, interaction networks, and substrate preference. These findings suggest their differentiated roles in regulating fatty acid composition in *S. wilsoniana*, particularly highlighting SwL8 as a promising candidate involved in linoleic acid metabolism.

## 5. Conclusions

In this study, a comprehensive strategy integrating fatty acid profiling, gene mining, structural modeling, and dynamics simulation was employed to identify key lipase genes potentially involved in fatty acid metabolism in *Swida wilsoniana*. Three candidates—SwL5, SwL8, and SwL12—were selected based on overall multi-ligand docking performance across the dominant fatty acids (including mean affinity across the three ligands) and substrate specificity toward dominant fatty acids. These lipases exhibited distinct structural features and dynamic behaviors, suggesting functional divergence in lipid accumulation. The results provide valuable insights into the molecular basis of oil composition in *S. wilsoniana*, and lay a theoretical foundation for future genetic improvement and functional characterization of oil-related genes in woody plants.

## Figures and Tables

**Table 1:** Binding Affinities Between Candidate Lipases and Three Major Fatty Acids.

Lipases	Linoleic Acid (kcal/mol)	Oleic Acid (kcal/mol)	Palmitic Acid (kcal/mol)	Mean Affinity Across Three Ligands (kcal/mol)
SwL1	−4.2	−3.1	−4.6	−3.97
SwL2	−5.6	−4.7	−4.5	−4.93
SwL3	−4.3	−5.4	−3.8	−4.50
SwL4	−4.7	−5.2	−3.5	−4.47
SwL5	−5.9	−6.1	−5.9	−5.97
SwL6	−6.6	−4.3	−4.5	−5.13
SwL7	−3.6	−2.9	−4.8	−3.77
SwL8	−7.3	−6.6	−5.4	−6.43
SwL9	−4.2	−4.6	−5.1	−4.63
SwL10	−5.8	−5.1	−3.4	−4.77
SwL11	−3.1	−4.6	−2.7	−3.47
SwL12	−6.4	−6.7	−3.8	−5.63
SwL13	−3.6	−4.4	−4.5	−4.17
SwL14	−4.3	−5.6	−5.2	−5.03
SwL15	−5.1	−5.1	−4.6	−4.93
SwL16	−2.8	−3.1	−5.3	−3.73
SwL17	−4.6	−5.3	−5.5	−5.13
SwL18	−5.4	−4.8	−4.7	−4.97

## Data Availability

The original contributions presented in this study are included in the article and the Supplementary Materials. The raw GC chromatograms and related output files, as well as the qPCR primer sequences, are provided in the Supplementary Materials. Further inquiries can be directed to the corresponding author.

## Supplementary Materials

The following supporting information can be downloaded at: https://www.mdpi.com/article/10.3390/biom16010092/s1, Table S1: Primers for qPCR.
