# Supplementary material for: Functional and Structural Insights into Lipases Associated with Fruit Lipid Accumulation in Swida wilsoniana
Source: Biomolecules. 2026 Jan 6;16(1):92. doi: 10.3390/biom16010092 (PMC12839255; doi:10.3390/biom16010092)
Supplement: Supplementary file 1 [file biomolecules-16-00092-s001.zip › Supplementary document.pdf]

Supporting Information

Functional and structural insights into lipases associated with fruit lipid accumulation in *Swida wilsoniana*

Wei Wu[a,b,1], Yunzhu Chen[a,1], Changzhu Li[a], Peiwang Li[a], Yan Yang[a], Lijuan Jiang[b], Wenyan Yuan[b], Qiang Liu[b], Li Li[a], Wenbin Zeng[b], Xiao Zhou[a,*], Jingzhen Chen[a,*]

[a] State Key Laboratory of Woody Oil Resources Utilization, Hunan Academy of Forestry, Changsha, Hunan 410004, PR China

[b] College of Life Science and Technology, Central South University of Forestry and Technology, 498 South Shaoshan Road, Changsha, Hunan 410004, PR China

Table S1 Primers for qPCR

Primers	Sequence(5′ →3′)
P_UBC	F：GATTGCATGGTGTGGAAA
	R：GTCTGTCCTATATGCATC
P_SwL5	F：GTTCAAATGACAATGGAGA
	R：TATGAATTAATTATAGTTA
P_SwL8	F：GCATCTTATGGACAGGTCG
	R：CTGGTATGTCAACTACTGC
P_SwL12	F：GCTAGAACTATGTTGTATA
	R：TTGGGCATATTAGAGCATTGC

>SwL5

MTMEIIGISLQPYSLFILLFNIVLSLHVFPCLSNFTAFVFGDSLVDAGNNDYLFT

LSKADSPPYGIDFKPSGGQPTGRFTNGRTISDLVGEALGGKSFPPPYLAPNSTS

NITLNGINYASGASGILDETGTLFIGRVPLREQINYFEQSRTCIVNVMGENGTK

AFLKKAIFSLTIGSNDVLNYFQPSIPYFEEEKLPPDVFQDFMLSNFTMQLKRLH

DLGARKFVVSGVGPLGCIPFVRALHLVHNGKCSVKVNTLIQGYNKRLIKVIKD

LNRELGPEAVFIYANSYDLVKGIMRNYRQYGFENGDTPCCGGYFPPFMCFKG

GNANASSVLCDDRSKYVFWDAYHPTEAANIIIAESLVNGDRSVSSPINIRQLYN

YN

>SwL8

MDRSAVSATPAVASFLCIFLLLPAAKADGDFTGELNIHRRSPVNGLCAQLIVPS

GYPCKEYTIQTKDGYLLGLQRLSSRSVILGAQRGFPVLLQHGLFMAGDAWFL

DSVDQSLGFILADHGFDVWVGNVRGTRWSHGHTSLSEKDKEFWDWSWEEL

ALYDLAEMIHFVNLVTNSKIFVVGHSQGTIMSLAAFTQPDIVKMVEAAALLCP

ISYLEHISAQFVLRIVDVHLDQVILALGIHQLNFKSDVGAHILDSICDGHVDCN

DLLTSITGENCCFNNSRVDFYLEYEPHPSSSKNLNHLFQMIRKGTFAKYDYGIL

KNLKQYGQLKPPNFDLSRIPESLPIWMAYGGNDALADVMDVQHTLKDLQSK

PELLYLENYGHIDFLLSVKAKEDVYDSMIGFFKSRERSSSS

>SwL12

MLYMASSRVLSEFVLVVYLLWFTVLTGSVCSQLEAQVPCFFIFGDSLVDNGNN

NGMLTLARANYSPYGIDFPQGTTGRFTNGRTYVDILAQLLGFPNNIPPYARTR

GRALLRGANYASGASGIRDETGNNLGDHMSMNQQVANFGRTVQQISRYFRG

DYNAVNSYLSKCIFYSGMGSNDYLNNYFMPNFYSTGSDYTPKAFAAALLQDY

SRQLTELYNLGARKVVVTAVGQIGCIPYQLARYNNSNSNSRCNEHINNAIILFN

SGLRKLVQHFNKGQLPAAKFVFLDSYKSSKDLVLNAASYGFQVVDKGCCGV

GRNNGQITCLPLQQPCDDRRKYLFWDAFHPTEAANILLAKKSYTSKSQSYAYP

INIQQLAML
